# Inflammatory Bowel Disease in Vietnam: Insights From a Retrospective Study at Bach Mai Hospital

**DOI:** 10.1155/grp/1618547

**Published:** 2026-02-28

**Authors:** Nam Hoai Nguyen, Hien Thi-Thu Nguyen, Trang Thu Khuc, Yen Thi Lo, Ha Thi-Ngoc Doan, Hieu Van Nguyen, Long Cong Nguyen

**Affiliations:** ^1^ Gastroenterology and Hepatology Center, Bach Mai Hospital, Hanoi, Vietnam, bachmai.gov.vn; ^2^ University of Internal Gastroenterology Department, University of Medicine and Pharmacy, Hanoi National University (VNU), Hanoi, Vietnam, ugal.ro

**Keywords:** Crohn′s disease, inflammatory bowel disease, ulcerative colitis

## Abstract

**Introduction:**

Inflammatory bowel disease (IBD), including Crohn′s disease (CD) and ulcerative colitis (UC), is an emerging disease in Southeast Asian countries, including Vietnam. This study was conducted to characterize patients with IBD who were managed as inpatients.

**Populations and Methods:**

This is a retrospective study at the Gastroenterology and Hepatology Center, Bach Mai Hospital, from January 1, 2022 to December 31, 2024. Medical records from clinical information of all patients admitted for inpatient treatment were used.

**Results:**

There were 242 patients with IBD, of whom 143 had CD and 99 had UC. In patients with UC, disease extent was classified as E1 (24.0%), E2 (62.2%), and E3 (23.8%). For patients with CD, the disease location was L1 (9.1%), L2 (48.3%), L3 (32.9%), and L4 (2.1%), and the disease phenotype was B1 (49.0%), B2 (16.8%), B3 (34.2%), with 16.0% having perianal lesions. A total of 81.8% of patients were treated with the step‐up therapy; 18.2% were treated with the top‐down strategy, and there was a difference between CD and UC. After 12 months of follow‐up, 20% of patients required therapy escalation, including 18.2% with CD and 23.2% with UC.

**Conclusions:**

Inpatients with IBD at Bach Mai Hospital often present with severe manifestations and poor prognostic factors. Although biologic agents are increasingly used in CD, most patients are still managed with a step‐up approach due to limited access to advanced treatments.

## 1. Introduction

Inflammatory bowel disease (IBD), including Crohn′s disease (CD) and ulcerative colitis (UC), is an emerging disease in Southeast Asian countries, including Vietnam [1]. Currently, the highest annual incidence rates of IBD are in Europe at 24.3 per 100,000 people/year for UC and 12.7 per 100,000 people/year for CD, in North America at 19.2 per 100,000 people/year for UC and 20.2 per 100,000 people/year for CD, and in Asia and the Middle East at 6.3 per 100,000 people/year for UC and 5.0 per 100,000 people/year for CD [2, 3]. Epidemiological data indicate that Vietnam is in the second stage of the disease transition, characterized by a rising incidence [4, 5]. Lifestyle factors and genetic predisposition are believed by many authors to have a specific association with IBD [6]. In most cases, IBD is often a diagnosis of exclusion, after ruling out other related conditions such as bacterial infections, intestinal tuberculosis, lymphoma, parasitic infestations, and colorectal cancer [7]. In addition, extraintestinal manifestations such as involving the joints, skin, and psychological disorders are often the main reasons for hospital visits; however, these patients are frequently managed by other specialties other than gastroenterology [8].

Although IBD has long been the subject of research, to date, there is no single gold standard for diagnosing CD or UC. Diagnosis must be based on a combination of clinical symptoms, biochemical findings, imaging, endoscopy, and histopathological [7].

Currently, the goal of treating IBD is to achieve and maintain remission, limit complications, and improve patients′ quality of life. The two main treatment strategies are step‐up and top‐down. The choice of treatment strategy should be individualized for each patient, depending on disease activity, prognostic factors, availability of healthcare facilities, and, especially, the economic conditions and circumstances of each patient [7].

In Vietnam, data on IBD remain limited. This study was conducted to provide an overview of the clinical characteristics and management of IBD in Vietnam, aimed at raising awareness and alerting clinicians to the growing burden of the disease.

## 2. Methods

All IBD patients were diagnosed according to the ECCO guidelines [9]. Diagnosis was based on clinical features, endoscopic findings, histopathology, radiology, and laboratory tests, after excluding differential diagnoses such as infections, intestinal tuberculosis, and malignancies. Patients whose clinical signs or investigative findings that could not reliably rule out other diseases were excluded from the study.

This was a retrospective study conducted at the Gastroenterology and Hepatology Center, Bach Mai Hospital, from January 1, 2022 to December 31, 2024. Medical records from clinical information of all patients admitted for inpatient treatment were used. A total of 242 patients were confirmed to have IBD.

Step‐up and top‐down approaches are two initial treatment strategies for IBD patients. In the top‐down approach, biologic therapy is initiated as the first‐line treatment. Conversely, in the step‐up approach, patients begin with conventional agents such as 5‐aminosalicylic acid, corticosteroids, or immunosuppressants before escalating to biologic agents if disease control is inadequate.

Ethical approval was waived by the Institutional Review Board of Bach Mai Hospital with number 1530/QĐ‐BVBM.

## 3. Statistical Analysis

Patient data were entered into Epidata 4.6 software and processed on SPSS 26.0.

Descriptive and inferential analysis was applied to the statistical analysis of the data. The comparison of categorical variables was analyzed using the chi‐square test. The level of significance was set at *p* < 0.05.

## 4. Results

In our study, we analyzed 242 patients, of whom 59.1% had CD and 40.9% had UC. The mean age of the study population was 40.8 years, with the youngest patient patient aged 16 and the oldest aged 83. In CD patients, the mean age was 31 years (range, 7–70 years), whereas for those with UC, it was 46.1 years (15–83 years). Overall, 37.2% of IBD patients were newly diagnosed. The proportion of patients with a disease duration of more than 10 years was the lowest, accounting for 3.7%. Extraintestinal manifestations were observed in 15 patients (6.2%) (Table 1).

**Table 1:** Demographic and clinical features of patients with IBD (*n* = 242).

Characteristics	*n*(%)
Crohn′s disease	143 (59.1%)
Ulcerative colitis	99 (40.9%)
Age (mean [SD], min–max)	40.8 ± 16.2 (16 ÷ 83)
Age at diagnosis	37.2 ± 16.6 (3 ÷ 81)
Newly diagnosed	90 (37.2%)
Male	140 (57.9%)
Clinical symptoms	
Hematochezia	148 (61.2%)
Diarrhea	70 (28.9%)
Constipation	4 (1.7%)
Abdominal pain	134 (55.4%)
Weight loss	41 (16.9%)
Extraintestinal manifestations	15 (6.2%)
History of surgery	
No	172 (71.1%)
Intestinal perforation	19 (7.9%)
Intestinal obstruction	6 (2.5%)
Intestinal stenosis	2 (0.8%)
Intestinal perforation with leakage into the abdominal cavity	6 (2.5%)
Enterocutaneous fistula	9 (3.7%)
Enterovaginal fistula	1 (0.4%)
Anal fistula and anal abscess	20 (8.3%)

Among 143 patients with CD, the distribution of disease location was 9.1% L1 (ileal), 48.3% L2 (colonic), 32.9% L3 (ileocolonic), and 2.1% L4 (upper gastrointestinal). The distribution of disease behavior was 49.0% B1 (nonstricturing and nonpenetrating), 16.8% B2 (stricturing), and 34.2% B3 (penetrating), with 16.0% exhibiting perianal involvement (B3p). The age at diagnosis was classified as 14.0% A1 (< 17 years), 62.2% A2 (17–40 years), and 23.8% A3 (> 40 years). Among 99 patients with UC, the extent of disease was 24.0% E1 (proctitis), 62.2% E2 (left‐sided colitis), and 23.8% E3 (pancolitis).

Most patients were hospitalized due to at least one severe prognostic factor. Among them, 83.9% had at least one severe feature, 61.2% had disease onset before the age of 40, and 33.1% had a history of surgery.

In 242 IBD patients, 81.8% of patients were treated with the step‐up strategy, whereas 18.2% received top‐down therapy **(**Table 2 **)**. The rate of patients who received step‐up approach for CD and UC was 73.4% and 93.9%, respectively; this difference was statistically significant with *p* < 0.001.

**Table 2:** Severe prognostic factors of patients when hospitalized (*n* = 242).

Severe prognostic factors	*n*(%)
Age under 40 years	148 (61.2%)
History of surgery	80 (33.1%)
Complications include fistula and stenosis	40 (16.5%)
Anal involvement	45 (18.6%)
Crohn′s disease involving the small intestine or upper gastrointestinal tract	61 (25.2%)
Ulcerative colitis with extensive disease (E3)	46 (19.0%)
Presence of at least one factor	203 (83.9%)

Among the study population, 49 patients (20.2%) required treatment escalation **(**Table 3 **)**. Specifically, 18.2% belonged to the CD group and 23.2% to the UC group. Treatment switching occurred in seven cases: five patients were switched from adalimumab to infliximab, and two from infliximab to adalimumab **(**Table 4 **)**. A total of 51 patients required surgery due to complications such as fistula or stenosis—of these, 47 cases were CD and only 4 cases were UC.

**Table 3:** Initial treatment for IBD patients (*n* = 242).

Therapeutic approach	IBD (*n* = 242) *n*(%)	CD (*n* = 143) *n*(%)	UC (*n* = 99) *n*(%)	*p*
Step‐up	198 (81.8%)	105 (73.4%)	93 (93.9%)	< 0.001
Top‐down	44 (18.2%)	38 (26.6%)	6 (6.1%)	

**Table 4:** Management of IBD patient follow‐up 12 months (*n* = 242).

Characteristics		IBD (*n* = 242) *n*(%)	CD (*n* = 143) *n*(%)	UC (*n* = 99) *n*(%)
Change of treatment regimen	No change	184 (76.0%)	111 (77.6%)	73 (73.7%)
	Treatment escalation	49 (20.2%)	26 (18.2%)	23 (23.2%)
	Treatment de‐escalation	9 (3.8%)	6 (4.2%)	3 (3.0%)
Surgical intervention due to complications		51 (21.1%)	47 (32.9%)	4 (4.0%)

## 5. Discussion

IBD remains underrecognized and frequently misdiagnosed in Vietnam. In this study, patients with CD were diagnosed at a younger age than those with UC, with mean ages of 31 and 46.1 years, respectively. Interestingly, the proportion of newly diagnosed UC patients was higher than that of CD (46.0% vs. 28.1%). This may be explained by the more typical endoscopic features of UC, whereas the diagnosis of CD often requires exclusion of other conditions such as intestinal tuberculosis and lymphoma, which may delay confirmation. CD often involves transmural inflammation, which predisposes patients to complications such as fistulas, perforation, and intestinal obstruction. This explains why CD patients had a higher rate of surgical intervention (26.2%), most commonly for perforation and bowel obstruction. In contrast, UC primarily affects the mucosal layer, and its symptoms can easily be mistaken for functional bowel disorders such as irritable bowel syndrome, especially when lesions are mild or limited. Nevertheless, diagnostic delays persist due to overlapping symptoms and limited disease awareness among clinicians. Anal complications were also common in our cohort. Among 13 patients with anal fistula or abscess, 11 had CD and only 2 had UC, consistent with the known predilection of CD for perianal involvement. These findings align with international literature, where perianal manifestations are considered a hallmark of CD.

In our study, the relatively high rate of IBD‐related complications requiring surgical intervention raises important considerations in patient management (Table 2). A total of 70 patients had a history of surgery, intestinal perforation (19 cases), anal fistula and anal abscess (20 cases). These findings suggest that a significant proportion of patients in Vietnam still present with advanced or complicated disease at the time of diagnosis. In comparison, studies from the United States have reported that the prevalence of abdominal surgery among UC patients was 53% higher than in individuals without IBD (*p* < 0.05) [2]. The high complication and surgery rates among Vietnamese IBD patients indicate delayed diagnosis and limited treatment options. Improving early detection and expanding access to biologic therapy will be essential to reduce disease complications in the future.

The management of patients with IBD remains challenging. In our cohort of 242 patients, 81.8% were treated using the step‐up approach, whereas 18.2% received top‐down therapy (Table 3). In comparison, a 12‐month follow‐up study of 3611 IBD patients in Spain reported that 34% were treated with systemic corticosteroids, 25% with immunosuppressants, 15% with biologic agents, and 5.6% underwent surgery [8]. Some authors have suggested that initiating high‐intensity therapy—such as biologics or combination regimens—from the outset may improve disease control and prevent complications[7]. However, this treatment strategy may not be easily applicable in Vietnam, where socioeconomic constraints significantly influence therapeutic decisions. For IBD patients, in addition to disease severity, assessing risk factors for poor prognosis and subsequently stratifying patients are significant for management and selecting appropriate treatment regimens [10, 11]. Table 2 shows that 83.9% of patients had at least one factor indicating poor prognosis. Our study found that 34.0% of IBD patients required a step‐up in treatment, and there was no difference in the rate of treatment step‐up between the CD and UC groups. If previous treatment fails, patients will undergo a comprehensive evaluation to rule out concomitant infectious causes, especially CMV and *Clostridium difficile*, and to optimize the current medications in terms of route of administration and dosage[10, 12]. There is still no response (neither clinically nor on endoscopy); a step‐up in treatment is necessary. The change in treatment level is consistent with the recommendations of major gastroenterology associations worldwide, such as ECCO, the American Gastroenterological Society (ACG), the British Gastroenterological Society (SBG), and the Asia‐Pacific Gastroenterological Society, regarding upgrading treatment for IBD when initial treatment is ineffective [10, 13–15].

Up to one‐fifth of patients taking anti‐TNF drugs may not respond initially, and an additional 10%–15% lose response each year despite initially responding well to anti‐TNF drugs [16–18]. Causes of primary nonresponse or secondary loss of response include concomitant intestinal infection, insufficient drug concentrations, and the body′s immune response to repeated use of anti‐TNF drugs, leading to the formation of antidrug antibodies[16]. In our study, we recorded seven cases, representing 10.3% of the total number of patients taking biological drugs who had to switch medications due to loss of response (Table 4). According to the ACG 2018 and ECCO 2019, Ustekinumab is recommended for achieving remission in patients with moderate‐to‐severe CD who do not respond to anti‐TNF therapy[10, 12, 13, 16]. ECCO 2019 also recommends the use of Vedolizumab when patients fail to respond to anti‐TNF therapy. According to NH Nguyen et al., other anti‐TNFs may be used if there is a loss of response or intolerance to the initially chosen anti‐TNF[19]. However, this approach is still under further study. At our hospital, as well as many large hospitals in Vietnam, we currently do not measure the concentration of biological drugs. Therefore, for patients who have lost response to biological drugs, after ruling out or treating concomitant infections, they will be treated with higher doses or shorter dose intervals. If the treatment goal is still not achieved, the patient will switch to a different biological drug. During the study period, because no IL‐12/23 antagonists such as Ustekinumab or integrin antagonists such as Vedolizumab were available, patients who lost response to the initial anti‐TNF medication were switched to another anti‐TNF as second‐line treatment.

### 5.1. Limitations of the Study

This retrospective study of patient data from a single unit is not representative. Indicators for assessing disease severity, such as the Mayo and CDAI scores, have not been applied consistently, leaving no basis for evaluating patient severity during retrospective data review. This is not only a management challenge at our hospital but also at many other hospitals in Vietnam.

## 6. Conclusion

In Vietnam, patients with IBD are often hospitalized with severe disease or poor prognostic factors such as early onset, a history of surgery, and extensive gastrointestinal lesions on endoscopy. Although biologic therapy is increasingly used, the majority of CD patients are still managed with a step‐up approach due to economic and accessibility limitations. Early recognition, multidisciplinary management, and broader access to advanced therapies are essential to improve outcomes and reduce disease complications.

## Author Contributions

Long Cong Nguyen, Trang Thu Khuc, Yen Thi Lo, Hien Thi‐Thu Nguyen, and Nam Hoai Nguyen designed the study and collected and analyzed the data. Ha Thi‐Ngoc Doan and Hieu Van Nguyen helped with data collection and analysis. Long Cong Nguyen and Nam Hoai Nguyen contributed to the study design and helped interpret the results. All authors contributed to writing the manuscript.

## Funding

No funding was received for this manuscript.

## Ethics Statement

Ethical approval was waived by the Institutional Review Board of Bach Mai Hospital with number 1530/QĐ‐BVBM.

## Consent

The hospital does not require a consent form for retrospective data.

## Conflicts of Interest

The authors declare no conflicts of interest.

## Data Availability

The datasets generated and/or analyzed during the current study are available from the corresponding author upon reasonable request.
